# The Need for Improved Management of Painful Diabetic Neuropathy in Primary Care

**DOI:** 10.1155/2016/1974863

**Published:** 2016-03-02

**Authors:** Teresa Sobhy

**Affiliations:** McMaster University, Hamilton, ON, Canada L8S 4L8

## Abstract

The provision of care for patients with type II diabetes in primary care must involve assessing patients for peripheral neuropathy of the feet.* Objectives.* This paper will demonstrate that painful diabetic neuropathy (PDN) is poorly assessed for and treated in primary care.* Methods.* A critical analysis of research will be conducted to identify the prevalence and impact of PDN among individuals with type II diabetes.* Results.* Research evidence and best practice guidelines are widely available in supporting primary care practitioners to better assess for and treat PDN. However, the lack of knowledge, awareness, and implementation of such research and guidelines prevents patients with PDN from receiving appropriate care.* Discussion.* Much international research exists on the prevalence and impact of PDN in primary care; however, Canadian research is lacking. Furthermore, the quantity and quality of research on treatment modalities for PDN are inadequate. Finally, current research and guidelines on PDN management are inadequately implemented in the clinical setting.* Conclusion.* The undertreatment of PDN has significant implications on the individual, family, and society. Healthcare practitioners must be more aware of and better implement current research and guidelines into practice to resolve this clinical issue.

## 1. Background

Care of the diabetic patient in the primary care setting involves counseling the patient on diet, exercise, and medications to optimize diabetes self-management. The provision of diabetes care in the primary care setting must also involve assessing the patient for any signs and symptoms of diabetes-related complications. One of these complications is peripheral neuropathy of the feet. Patients with diabetic neuropathy often present with numbness, reduced sensation, and significant levels of pain in the feet. Neuropathic pain can be defined as “pain arising as a direct consequence of a lesion or disease affecting the somatosensory system” [1].

## 2. Objectives

This paper will discuss the undertreatment of painful diabetic neuropathy (PDN) in primary care. Associated implications on the individual, family, and society in failing to treat PDN will be discussed, along with suggestions for future research and clinical practice.

## 3. Methods

A literature search was conducted to identify the prevalence and impact of undertreated PDN among individuals with type II diabetes, particularly among those receiving care in the primary care setting.

## 4. Results

A primary care-based study done in the United Kingdom (UK) by Haanpää and colleagues [2] indicated that 26% of type II diabetics experience PDN. A study done in China on 1193 type II diabetic outpatients identified diabetic neuropathy in 17% of participants; thirty-two percent of these patients with known neuropathy had not received any treatment [3]. Van Acker and colleagues [4] conducted a cross-sectional study of 1111 patients with type I (*n* = 344) and type II (*n* = 767) diabetes in outpatient clinics. Researchers in this study found that, of the patients suffering from PDN, less than one-third (28%) were receiving recommended treatments for neuropathic pain such as antidepressants/anticonvulsants. A United States-based study of 7892 type II diabetics enrolled from 2142 sites found a 37% prevalence of neuropathy within the sample [5]. This 37% prevalence was detected through the use of the 10 gram monofilament, while diagnosis of neuropathy based on physician perception alone was only 14% [5]. This study identified that physicians have a tendency to underestimate neuropathy in patients when using perception alone (rather than a diagnostic tool) and consequently undertreat this complication. Individuals with neuropathic pain also report significantly less effective pain relief than individuals suffering from nonneuropathic pain [6]. Neuropathic pain is underrecognized and undertreated in primary care despite the availability of effective pharmaceutical treatments. Primary care practitioners are not only failing to diagnose PDN in a timely manner but are also late in initiating pharmacotherapy for these patients [2]. One reason for this as suggested by Hovaguimian and Gibbons [7] is due to the challenges faced by practitioners in selecting an appropriate agent for treatment, given the extent of available options. Choosing the wrong treatment or delaying treatment for patients suffering from PDN can worsen their condition [2]. These studies indicate that PDN is significant in its prevalence and pain severity and yet remains underrecognized, undertreated and consequently unrelieved among type II diabetics.

### 4.1. Impact of Underrecognized and Undertreated PDN

The inadequate treatment of type II diabetics suffering from PDN poses a significant impact on the individual, family, and society. On the individual level, type II diabetics with PDN are significantly more likely to experience lower quality of life in comparison to patients with other types of pain such as nociceptive pain [2]. PDN has also been shown to contribute to anxiety, depression, and reduced sleep quality [8]. Lalli and colleagues [9] demonstrated that patients with PDN were at a greater risk of fall-related injuries and hospitalizations. In considering the impact of PDN on society, research has shown that PDN is linked to work-related disability and increased healthcare costs [10]. The increased risk of fall-related injuries and hospitalizations also has financial implications for the healthcare system. Work disruptions, injuries, and psychosocial impacts of pain (anxiety, depression, etc.) can also impact the family of the PDN sufferer. Finally, the burden of neuropathic pain has been shown to rise with pain severity—activity levels, work disruptions and healthcare utilization are more greatly impacted with increased pain related to neuropathy [11].

### 4.2. Diagnosing Painful Diabetic Neuropathy

The Canadian Diabetes Association (CDA) provides people with diabetes as well as healthcare professionals with supportive resources to optimize diabetes management. Its best practice guidelines are published every five years and represent “the best and most current evidence-based clinical data for healthcare professionals” [12]. The CDA identifies two options for screening for peripheral neuropathy of the feet including the 10 gram monofilament sensitivity test and sensitivity to vibration of the dorsum of the great toe [12]. These methods of screening have demonstrated reliability in diagnosing PDN and should be used by primary care practitioners as part of their regular diabetic patient assessments. Rahman et al. [13] discuss how the 10 gram monofilament test is the most accurate and feasible tool to use in primary care to diagnose peripheral neuropathy. More recently, Kanji et al. [14] study found that abnormal results on monofilament testing is one of the most helpful signs in diagnosing peripheral neuropathy. The vibration test was considered the “gold standard” in this study but it is more complex and less feasible for regular use in primary care. The 10 gram monofilament test was shown to be both accurate and feasible to implement in primary care [14]. Perkins et al. [15] conducted a longitudinal cohort study of 175 type II diabetics to determine the accuracy of the 10 gram monofilament examination to predict the incidence of diabetic neuropathy over four years. These researchers found that this tool was effective in predicting the risk of future neuropathy through a defined scoring system [15].

### 4.3. Treating Painful Diabetic Neuropathy

In order to manage risks associated with PDN, patients should be treated with appropriate pain medication such that pain is reduced by 50% in four weeks' time [16]. With respect to treating PDN, the CDA [12] provides specific guidelines and a treatment algorithm for practitioners to follow in treating PDN. The guidelines include important and relevant information such as different classes of medications that can be used (anticonvulsants, opioids, and others), suggested starting doses, titration schedules, maximum doses, and even estimated monthly costs of each of the listed medications. These guidelines provide valuable information to the prescriber on diagnosis of PDN and support the practitioner in providing adequate and effective pharmaceutical treatments to these patients.

## 5. Discussion

### 5.1. Implications for Research

Limited research has been conducted on the prevalence and significance of underrecognized and undertreated PDN in Canada. As previously mentioned, the majority of studies found were conducted in the United States and Europe. Therefore, it can be argued that there is a need for more research to be done in the primary care setting in Canada to gain a better understanding of PDN screening, diagnosis, and treatment, along with relevant recommendations to Canadian primary care practitioners. While research from other countries remains relevant, it is useful for Canadian practitioners to be aware of the significance and impact of this issue among local primary care settings.

The significance and prevalence of undertreated PDN is discussed in detail in current research, but solutions to the problem are not adequately addressed. Research on this issue should provide more guidance to primary care practitioners on how to better assess for PDN and appropriate treatment options at diagnosis and for ongoing care. The validated tools for assessing PDN, such as the 10 gram monofilament test, should also be emphasized for use by practitioners in research studies that aim to highlight and resolve this clinical issue.

With respect to research regarding treatment options, a 2011 report from the American Academy of Neurology (AAN) demonstrated that there are an insufficient number of high-quality comparative drug therapy studies on PDN. The studies that do exist lack consistency in their use of pain measurement tools. The AAN [17] recommends a formalized approach for rating pain across clinical trials that evaluate treatment modalities for PDN. For future studies, the AAN [17] also recommends studying patient quality of life and physical function when assessing the effectiveness of treatment interventions. Studies on PDN also often fail to identify cost effectiveness in terms of pharmacotherapy, which is of relevance to the primary care practitioner in making treatment decisions [17]. This report also identifies that there are very few studies that directly compare different treatment modalities for PDN, and this is a future consideration for research in this area [17]. Future studies of various pharmacotherapies should also be carried out over a longer duration. Recently conducted studies are of relatively short duration and may not be sufficient in capturing the long-term effects of these treatment modalities; this is important to consider as PDN is a chronic condition [17]. The AAN [17] also recommends putting forth more research around the use of electrical stimulation and its effects on PDN as there is currently poor understanding of its role and effectiveness as a potential treatment option.

Finally, while the literature provides compelling statistics on the undertreatment of PDN in primary care, there is an overall lack of a guiding framework or theory to implement changes in the practice setting. Current research provides little on how to implement practice changes across the primary care setting to address and minimize this clinical issue.

### 5.2. Implications for Clinical Practice

The issue of the undertreatment of PDN in type II diabetics has several important implications for clinical practice in primary care. Primary care practitioners must be educated on the prevalence of PDN among type II diabetics, as well as on how to diagnose and treat PDN in a timely manner. The CDA Clinical Practice Guidelines [18] provide comprehensive guidelines on the assessment and treatment of PDN. The guidelines explicitly state that “the under-diagnosis of neuropathy is a fundamental problem in primary care…and impedes the benefits of early identification, the management necessary to achieve improved glycemic control and the prevention of neuropathy related sequelae” [12]. Evidently, there exists a gap in primary care practitioners' knowledge and application of these important guidelines. Smith et al.'s [8] research points to the lack of education and resources available for practitioners as the reason for undertreatment of PDN. For primary care practitioners in Canada, the resources are clearly available for treating PDN, but the key issue seems to be the lack of education/awareness of the prevalence and significance of PDN, the importance of treating it, and the practice guidelines available for support in doing so.

## 6. Conclusion

Painful diabetic neuropathy is a complication of type II diabetes that is significant in its prevalence and impact which extends beyond the pain experienced by the individual sufferer. In Canada, the CDA provides evidence-based guidelines that are based on the most current research in the field. These guidelines provide comprehensive education and support for primary care practitioners in the assessment and treatment of PDN. However, these guidelines are underutilized and thus type II diabetics with PDN remain undertreated in primary care. The use of a simple 10 gram monofilament test can facilitate more timely diagnoses and treatments for patients with PDN.

There is a need for greater awareness and education around the impact of PDN and the availability of comprehensive resources to aid primary care practitioners in managing and treating PDN in diabetic patients. This paper demonstrated the significance and impact of underrecognized and undertreated PDN in the primary care setting. The literature search on this clinical issue was discussed and analyzed, along with implications for future research and clinical practice. With appropriate diagnosis, treatment, and follow-up, the number of patients suffering from PDN can be reduced and thus its detrimental implications will be alleviated.
